# Supplementary material for: Analysis of 1-Aroyl-3-[3-chloro-2-methylphenyl] Thiourea Hybrids as Potent Urease Inhibitors: Synthesis, Biochemical Evaluation and Computational Approach
Source: Int J Mol Sci. 2022 Oct 1;23(19):11646. doi: 10.3390/ijms231911646 (PMC9570211; doi:10.3390/ijms231911646)
Supplement: Supplementary file 1 [file ijms-23-11646-s001.zip › ijms-1935362-supplementary.pdf]

SUPPLEMENTARY FILE

Analysis of 1-aroyl-3-[3-chloro-2-methylphenyl] thiourea hybrids as potent urease inhibitors: Synthesis, biochemical evaluation and computational approach

Samina Rasheed [1], Mubashir Aziz [2], Aamer Saeed [1,*], Syeda Abida Ejaz [2,*], Pervaiz Ali Channar [1,3], Seema Zargar [4], Qamar Abbas [5], Humidah Alanazi [4], Mumtaz Hussain [6], Mona Alharbi [4], Song Ja Kim [7], Tanveer A. Wani [8] and Hussain Raza [7]

1 Department of Chemistry, Quaid-i-Azam University, Islamabad 45320, Pakistan
2 Department of Pharmaceutical Chemistry, Faculty of Pharmacy, The Islamia University of Bahawalpur, Bahawalpur 63100, Pakistan
3 Institute of Chemistry, Shah Abdul Latif University, Khairpur 66020, Pakistan
4 Department of Biochemistry, College of Science, King Saud University, Riyadh 11451, Saudi Arabia
5 Department of Biology, College of Science, University of Bahrain, Sakhir 32038, Bahrain
6 Department of Chemistry, University of Karachi, Karachi 75270, Pakistan
7 Department of Biological Sciences, College of Natural Sciences, Kongju National University, Gongju 32588, Korea
8 Department of Pharmaceutical Chemistry, College of Pharmacy, King Saud University, P.O. Box 2457, Riyadh 11451, Saudi Arabia
* Correspondence: aamersaeed@yahoo.com (A.S.); abidaejaz2010@gmail.com (S.A.E.); Tel.: +92-062-9250245 (S.A.E.)

Citation: Rasheed, S.; Aziz, M.; Saeed, A.; Ejaz, S.A.; Channar, P.A.; Zargar, S.; Abbas, Q.; Alanazi, H.; Hussain, M.; Alharbi, M.; et al. Analysis of 1-Aroyl-3-[3-chloro-2-methylphenyl] Thiourea Hybrids as Potent Urease Inhibitors: Synthesis, Biochemical Evaluation and Computational Approach. *Int. J. Mol. Sci.* **2022**, *23*, 11646. https://doi.org/10.3390/ijms231911646

Academic Editor: Małgorzata Borówko

Received: 9 September 2022
Accepted: 26 September 2022
Published: 1 October 2022

Publisher's Note: MDPI stays neutral with regard to jurisdictional claims in published maps and institutional affiliations.

Copyright: © 2022 by the authors. Licensee MDPI, Basel, Switzerland. This article is an open access article distributed under the terms and conditions of the Creative Commons Attribution (CC BY) license (https://creativecommons.org/licenses/by/4.0/).

Experimental

Characterization data

4a) N-((3-chloro-2-methylphenyl)carbamothioyl)benzamide

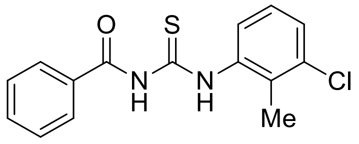

Off white powder, Yield=82%, M.P=134°C, R_f =0.25(n-Hexane: Ethyl acetate 1:1), FTIR(cm^{-1}): 1248 (C=S stretch), 3241 (N-H stretch), 2979.4 (Aromatic C-H, stretching), 1582 (C-C, stretching), 1138 (C-N stretch), ^{1}H-NMR (300 MHZ, DMSO-d6): δ (ppm) 12.161 (s, 1H, ArCO-N-H),11.815 (s,1H,Ar-NH), 8.037-7.609 (m,4H,Ar), 7.448-7.290(m.3H,Ar), 1.62 (s, 3H, CH3) 13C-NMR (75 MHz, DMSO-d6) 181.06 (C=S), 167.60(C=O), 139.25, 138.49, 134.23, 133.18, 131.41, 131.21, 131.08, 129.00, 128.44, 127.57, 127.12, 15.57(CH_3); Anal. Calcd. for $C_{16}H_{15}ClN_2O_2S$, C, 57.40; H, 4.52; N, 8.37; S, 9.58, found; C, 58.41; H, 4.63; N, 8.71; S, 9.62.

4b) N-((3-chloro-2-methylphenyl)carbamothioyl)-4-methoxybenzamide

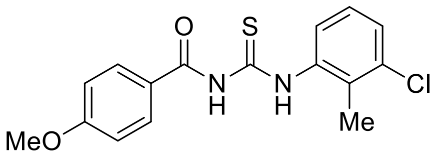

Off white crystalline powder, Yield: 85%, M.P=145°C, R_f = 0.28 (n-Hexane: Ethyl acetate 1:1),FTIR(cm^{-1}): 1238 (C=S stretch), 3256 (N-H stretch), 2978.8 (Aromatic C-H, stretching),1572 (C-C, stretching), 1132 (C-N stretch), ^{1}H-NMR (300 MHZ, DMSO-d6): δ (ppm) 12.350(s, 1H, ArCO-N-H), 9.207 (s,1H,Ar-NH), 7.916-6.983 (m, 7H, Ar), 3.914(s, 3H, OCH_3), 2.398 (s, 3H, CH_3 13C-NMR (75MHz, DMSO-d6) δ(ppm): 180.17 (C=S), 166.54(C=O), 164.1, 137.7,135.2, 132.5, 129. 8,127.0, 126.7, 125.4, 114.5, 144.0, 55.6(OCH_3), 15.23 (CH_3); Anal. Calcd. for $C_{15}H_{13}ClN_2OS$, C, 46.95; H, 3.15; N, 7.30; S, 8.36, found; C, 45.99; H, 3.44N, 7.20; S, 8.23.

4c) N-((3-chloro-2-methylphenyl)carbamothioyl)-3,5-dimethoxybenzamide

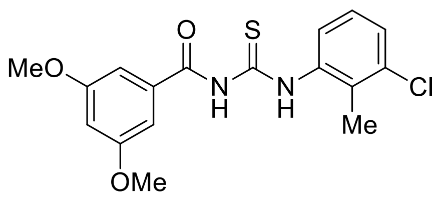

Colourless crystals, Yield: 83%, M.P=152°C, R_f = 0.31(n-Hexane: Ethyl acetate 1:1), FTIR(cm^{-1}): 1236(C=S stretch), 3255 (N-H stretch), 2949.6 (Aromatic C-H, stretching), 1572 (C-C, stretching), 1135 (C-N stretch), ^{1}H-NMR (300 MHZ, DMSO-d6): δ (ppm) 12.020(s, 1H, ArCO-N-H),11.207 (s,1H,Ar-NH), 8.516-6.983 (m,6H,Ar), 3.614(s,6H,2(OCH_3)), 2.498 (s, 3H, CH3) 13C-NMR (75 MHz, DMSO-d6) : δ(ppm) 181.17 (C=S),169.0(C=O),161.8,140.3,137.4,134.7,125.7,124.8,124.5,104.0,103.8,55.9(OCH_3), 15.23 (CH_3); Anal. Calcd. for $C_{17}H_{17}ClN_2O_3S$ C, 55.96; H, 4.70; N, 7.68; S, 8.79, found; C, 56.16; H, 4.90; N, 7.65; S, 8.57.

4d) N-((3-chloro-2-methylphenyl)carbamothioyl)-4-methylbenzamide

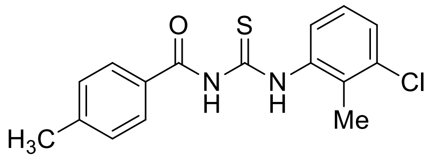

Light yellow powder, Yield=86%, M.P=128°C, R_f =0.31(n-Hexane: Ethyl acetate 1:1), FTIR(cm^{-1}): 1238(C=S stretch), 3259 (N-H stretch), 2947 (Aromatic C-H, stretching), 1572 (C-C stretching), 1145 (C-N stretch),

^{1}H-NMR (300 MHZ, DMSO-d6): δ (ppm) 11.99 (s, 1H, ArCO-N-H), 11.30 (s,1H,Ar-NH), 7.816-6.98 (m,7H,Ar), 2.61 (s, 3H, CH_3), 2.398 (s, 3H, CH_3), ^{13}C-NMR (75MHz, DMSO-d6): δ(ppm) 182.1 (C=S), 169.3 (C=O), 141.8, 140.3, 137.4, 134.7, 129.2, 127.4, 124.8, 124.4, 24.4, 6.1 Anal. Calcd. for $C_{16}H_{15}ClN_2OS$, C, 60.28; H, 4.74; N, 8.79; S, 10.06, found; C, 61.08; H, 4.81; N, 8.77; S, 10.23

4e) N-((3-chloro-2-methylphenyl)carbamothioyl)-4-nitrobenzamide

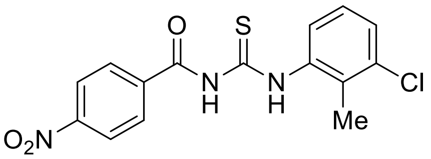

Light brown powder, Yield= 88%, M.P=122°C, R_f = 0.26 (n-Hexane: Ethyl acetate 1:1),FTIR(cm^{-}1): 1246(C=S stretch), 3299 (N-H stretch), 2947.4 (Aromatic C-H, stretching), 1572 (C=C), 1175 (C-N stretch), ^{1}H-NMR (300 MHZ, DMSO-d6): δ (ppm) 12.00 (s, 1H, ArCO-N-H), 11.707 (s, 1H, Ar-NH), 8.37 (d, 2H, Ar-NO2), 8.21 (d, 2H, Ar-NO2), 6.22-6.76 (m, 3H,Ar), 2.35 (s, 3H, CH3), ^{13}C-NMR (75MHz,DMSO-d6): δ(ppm) 180.1 (C=S), 168.3 (C=O),151.8,140.3,137.8,134.7,128.4,127.5,124.8,124.5,121.2,6.7 (CH_3) Anal. Calcd. for $C_{15}H_{12}ClN_3O_3S$, C, 51.51; H, 3.46; N, 12.01; S, 9.17, found; C, 51.61; H, 3.51; N, 12.61; S, 9.77.

4f) N-((3-chloro-2-methylphenyl)carbamothioyl)-3-nitrobenzamide

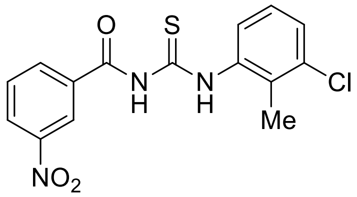

Light yellow, Yield=87%, M.P=132°C, R_f =0.29 (n-Hexane: Ethyl acetate 1:1), FTIR(cm^{-}1): 1226(C=S stretch), 3229 (N-H stretch), 2957.4 (Aromatic C-H, stretching), 1572 (C=C), 1155 (C-N stretch), ^{1}H-NMR (300 MHZ, DMSO-d6): δ (ppm) 12.20(s, 1H, ArCO-N-H),11.607 (s,1H,Ar-NH),8.37-7.44 (m,4H,Ar-NO2),7.32-6.76(m.3H,Ar), 2.45(s, 3H, CH3), ^{13}C-NMR (75 MHz, DMSO-d6) : δ(ppm) 180.3(C=S), 167.3(C=O),148.5,140.3,137.4,135.2, 133.6,139.8, 127.5, 124.5, 124,3, 122.4,6.8(CH_3), Anal. Calcd. for $C_{15}H_{12}ClN_3O_3S$, C, 51.51; H, 3.46; N, 12.01; S, 9.17 found C, 52.71; H, 3.66; N, 12.41; S, 9.67.

4g) N-((3-chloro-2-methylphenyl)carbamothioyl)-3,5-dinitrobenzamide

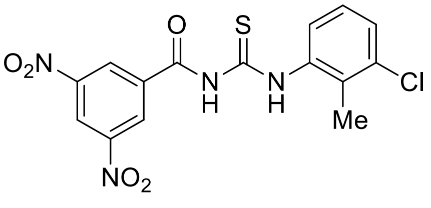

White crystals, Yield= 78%, M.P =124°C, R_f = 0.27 (n-Hexane: Ethyl acetate 1:1), FTIR(cm^{-}1): 1232(C=S stretch), 3226 (N-H stretch), 2977.4 (Aromatic C-H, stretching), 1572 (C=C), 1115 (C-N stretch), ^{1}H-NMR (300 MHZ, DMSO-d6): δ (ppm) 12.70(s, 1H, ArCO-N-H),11.577 (s,1H,Ar-NH),8.27-7.47 (m,3H,Ar-NO2),7.32-6.36(m.3H,Ar), 2.46 (s, 3H, CH3), ^{13}C-NMR (75 MHz, DMSO-d6) : δ(ppm)178.3 (C=S), 166.3(C=O), 149.4, 140.3, 137.4, 136.0, 134.7, 128.5, 127.5, 124.8, 124.4, 122.1, 15.23 (CH_3) Anal.Calcd. for $C_{15}H_{11}ClN_4O_5S$, C, 45.63; H, 2.81; N, 14.19; S, 8.12 found; C, 46.33; H, 2.91; N, 14.29; S, 8.16.

4h) 4-chloro-N-((3-chloro-2-methylphenyl)carbamothioyl)benzamide

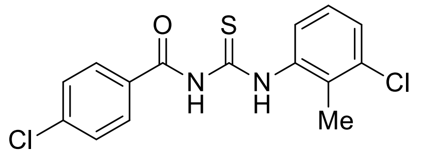

Light yellow crystals, Yield=75%, M.P=160°C, R_f = 0.25 (n-Hexane: Ethyl acetate 1:1), FTIR (cm^{-1}): 1242(C=S stretch), 3227 (N-H stretch), 2979.4 (Aromatic C-H, stretching), 1575(C-C stretching), 1115 (C-N stretch), ^{1}H-NMR (300 MHZ, DMSO-d6); δ (ppm) 11.90 (s, 1H, ArCO-N-H), 11.537 (s, 1H, Ar-NH), 8.37-7.57 (d, 2H, Ar-Cl), 7.52-6.96 (d, 2H,Ar-Cl), 6.72-6.53(m,3H,Ar), 2.96 (s, 3H, CH3), ^{13}C-NMR (75 MHz, DMSO-d6) : δ(ppm) 185.3(C=S), 168.3(C=O), 140.3, 137.7, 137.4, 134.7, 132.3, 129.0,128.9, 127.5, 124.8, 124.5, 15.24(CH$_3$)Anal. Calcd. for C15H12Cl2N2OS; C, 53.11; H, 3.57; N, 8.26; S, 9.45 found; C, 53.13; H, 3.77; N, 8.36; S, 9.85.

4i) 2-chloro-N-((3-ethyl-2-methylphenyl)carbamothioyl)benzamide

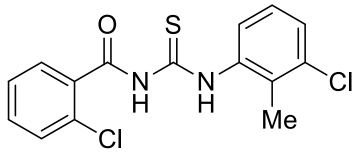

Light yellow precipitates, Yield=80%, M.P=150°C, R_f = 0.25 (n-Hexane : Ethyl acetate 1:1), FTIR (cm^{-1}): 1222(C=S stretch), 3226 (N-H stretch), 2996.4 (Aromatic C-H, stretching), 1565 (C-C, Stretching), 1125 (C-N stretch), ^{1}H-NMR (300 MHZ, DMSO-d6); δ (ppm) 12.00(s, 1H, ArCO-N-H), 11.837 (s,1H,Ar-NH), 8.37-7.57 (m,4H,Ar-Cl), 7.52-6.96(m.3H,Ar), 2.36 (s, 3H, CH3), ^{13}C-NMR (75 MHz, DMSO-d6) : δ(ppm)181.3(C=S), 169.3(C=O), 140.3, 137.4, 134.7, 133.6 ,132.4, 132.2, 129, 129.9, 127.9, 127 ,124.8, 124.5, 15.25(CH$_3$), Anal. Calcd. for $C_{15}H_{12}Cl_2N_2OS$; C, 53.11; H, 3.57; N, 8.26; S, 9.45 found: C, 53.23; H, 3.71; N, 8.37; S, 10.12.

4j) 2, 4-dichloro-N-((3-chloro-2-methylphenyl)carbamothioyl)benzamide

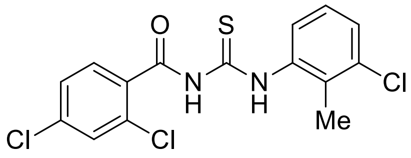

White precipitates, Yield = 82%, M.P =122°C, R_f =0.26, (n-Hexane: Ethyl acetate 1:1), FTIR y (cm1)1242 (C=S stretch), 3236 (N-H stretch), 2986.4 (Aromatic C-H, stretching), 1566 (C-C, Stretching), 1128 (C-N stretch), ^{1}H-NMR (300 MHZ, DMSO-d6); δ (ppm) 12.10 (s, 1H, ArCO-N-H), 11.937 (s, 1H, Ar-NH), 8.47-7.67 (m, 3H, Ar-Cl), 7.53-6.76(m, 3H, Ar), 2.39 (s, 3H, CH3), ^{13}C-NMR (75MHz, DMSOd_6) δ(ppm) 182.3 (C=S), 166.3 (C=O), 140.3, 139.1, 137.4, 134.7, 133.7,133.7, 130.5, 130.4, 130.3, 127.5, 124.8, 124.5, 15.26 (CH$_3$); Anal. Calcd. C, 48.21; H, 2.97; N, 7.50; S, 8.58 found: C, 48.23; H, 2.89; N, 7.67; S, 8.62.

Supplementary images

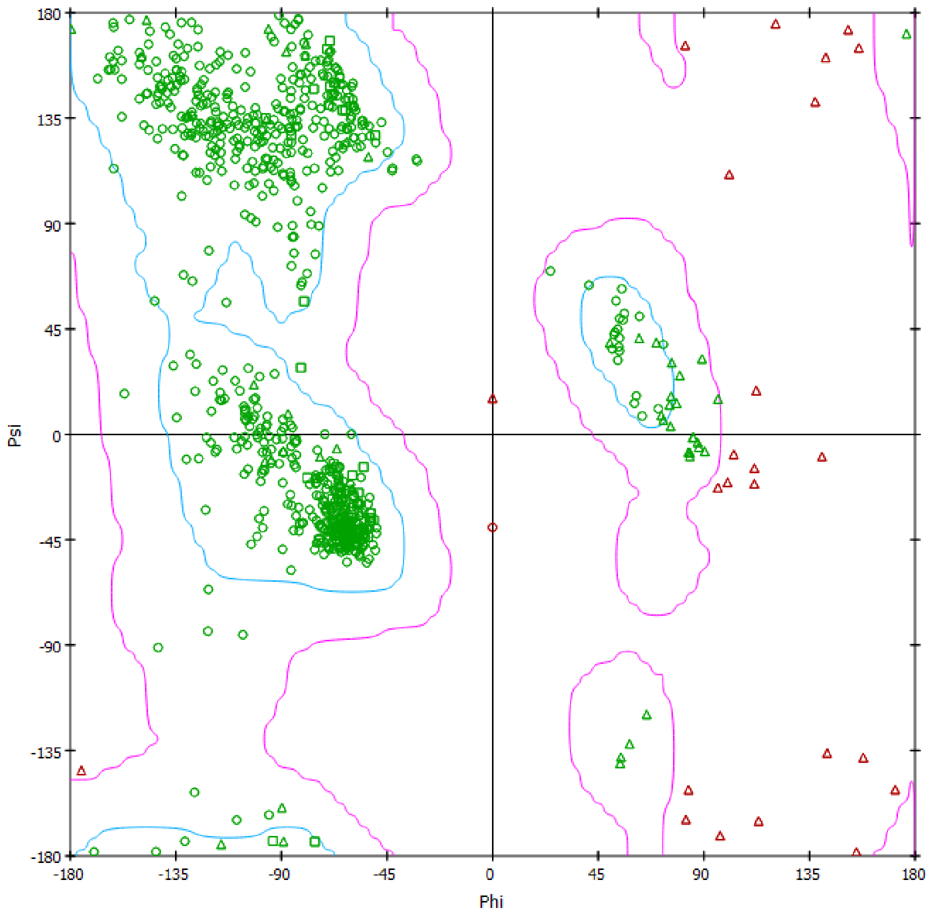

Figure S1. Ramachandran graph of jack bean urease

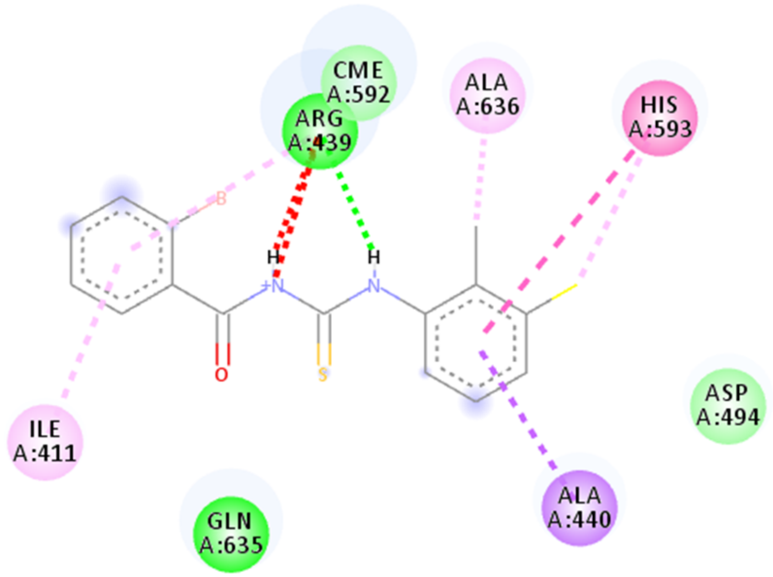

Figure S2. Docking complex **4a**

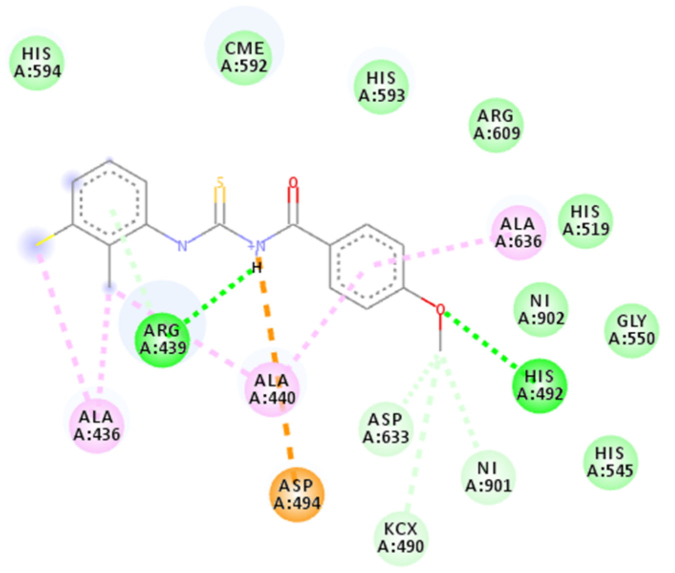

Figure S3. Docking complex **4b**

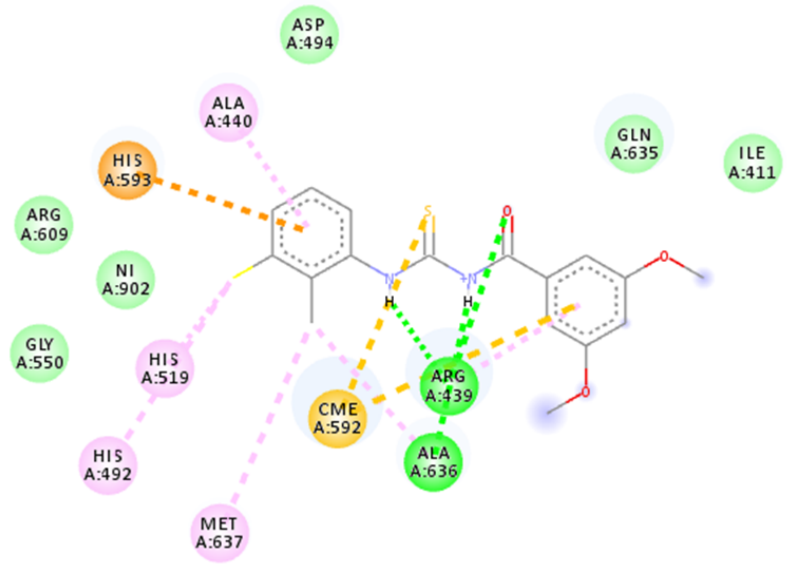

Figure S4. Docking complex **4c**

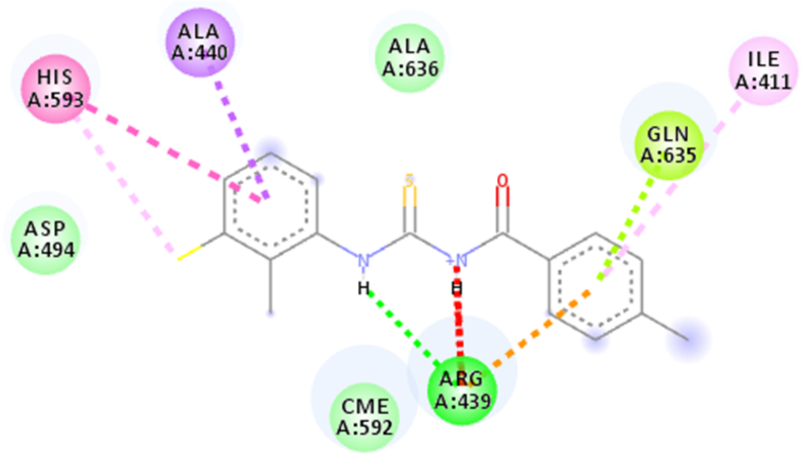

Figure S5. Docking complex **4d**

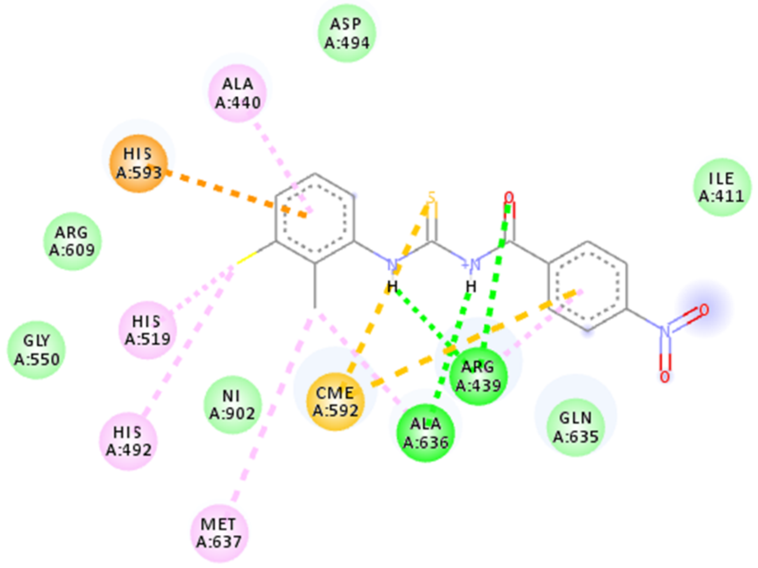

Figure S6. Docking complex **4e**

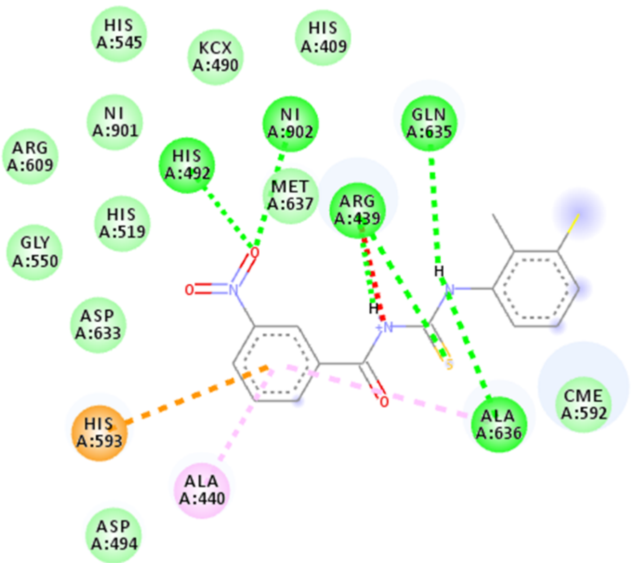

Figure S7. Docking complex **4f**

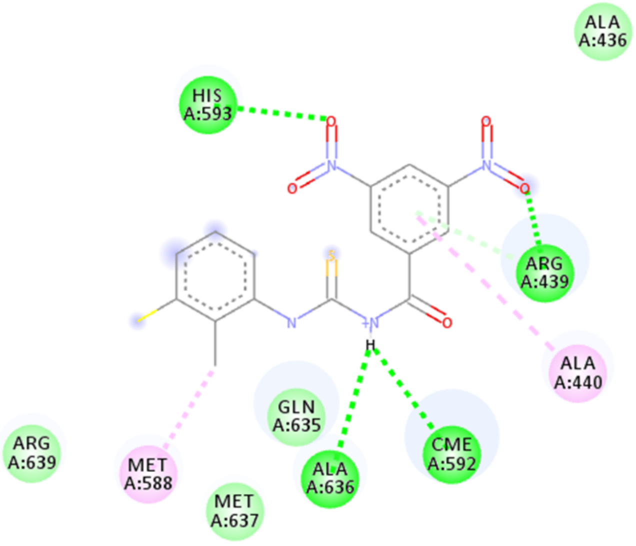

Figure S8. Docking complex **4g**

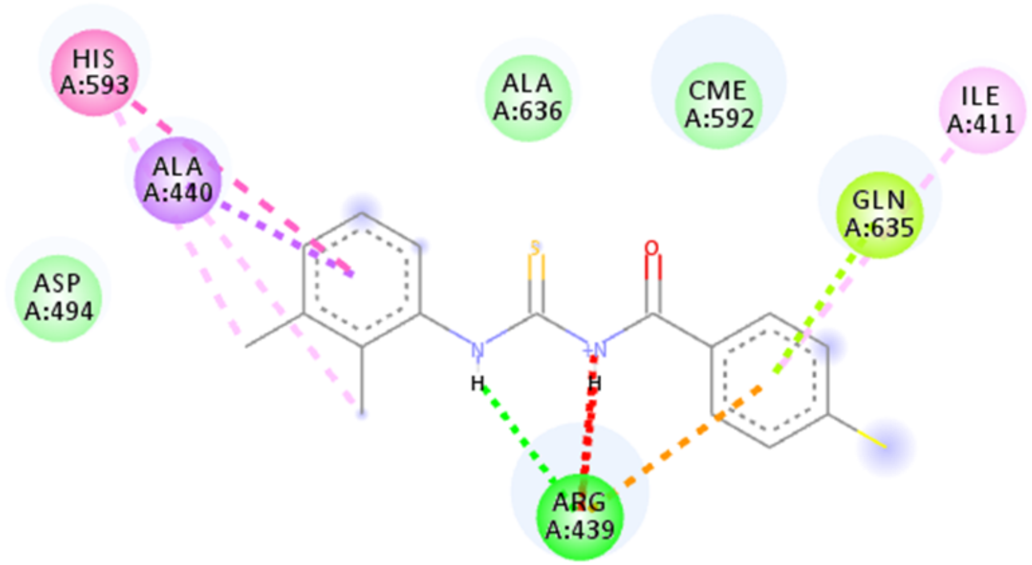

Figure S9. Docking complex **4h**

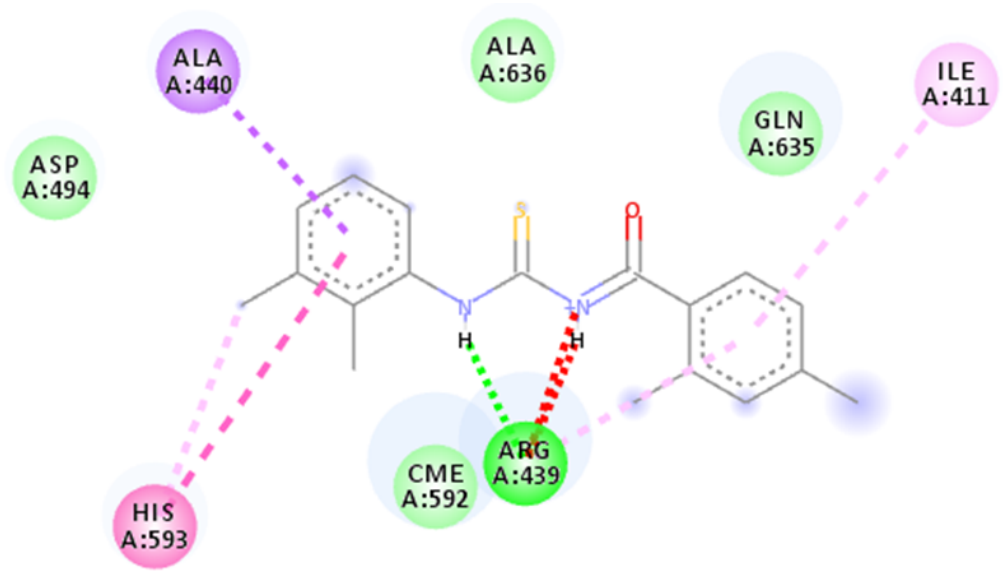

Figure S10. Docking complex **4j**

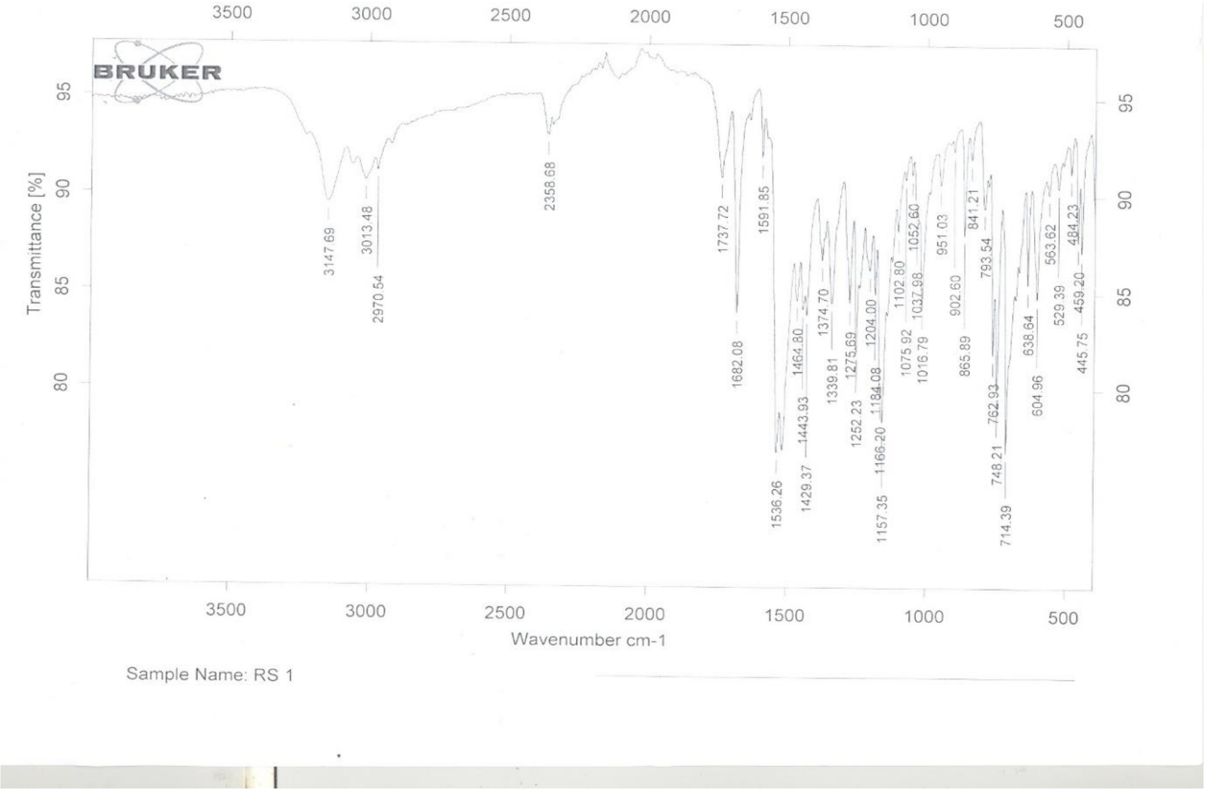

Figure S11 IR Spectrum of **4a**

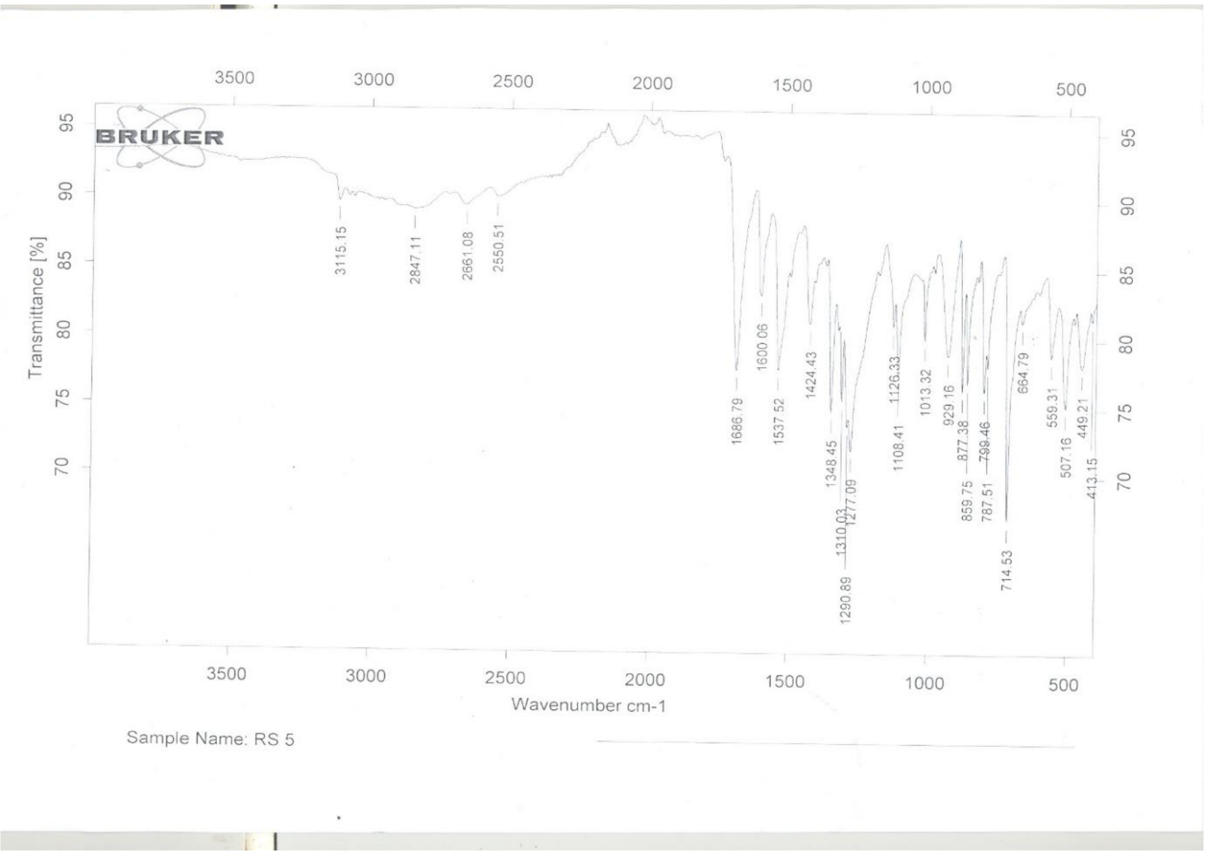

Figure S12. IR Spectrum of **4e**

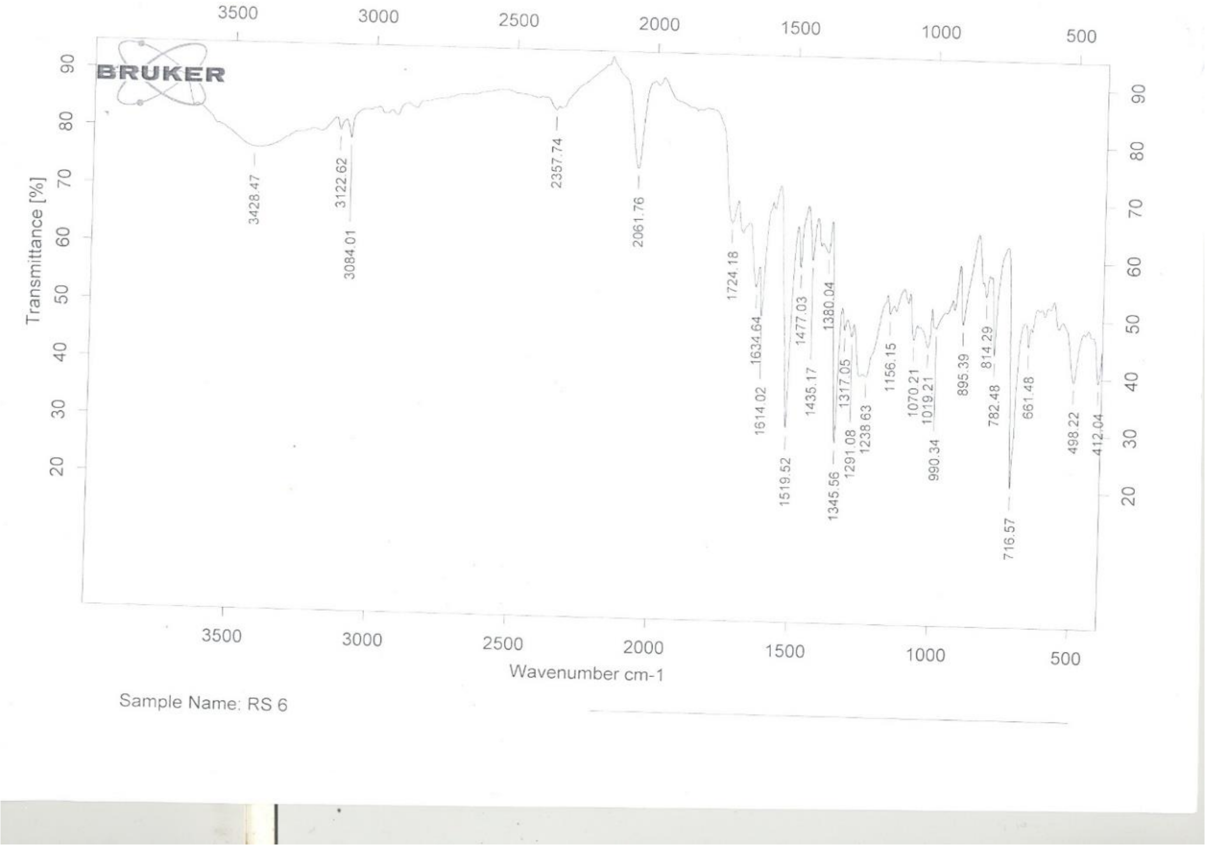

Figure S13. IR Spectrum of **4f**

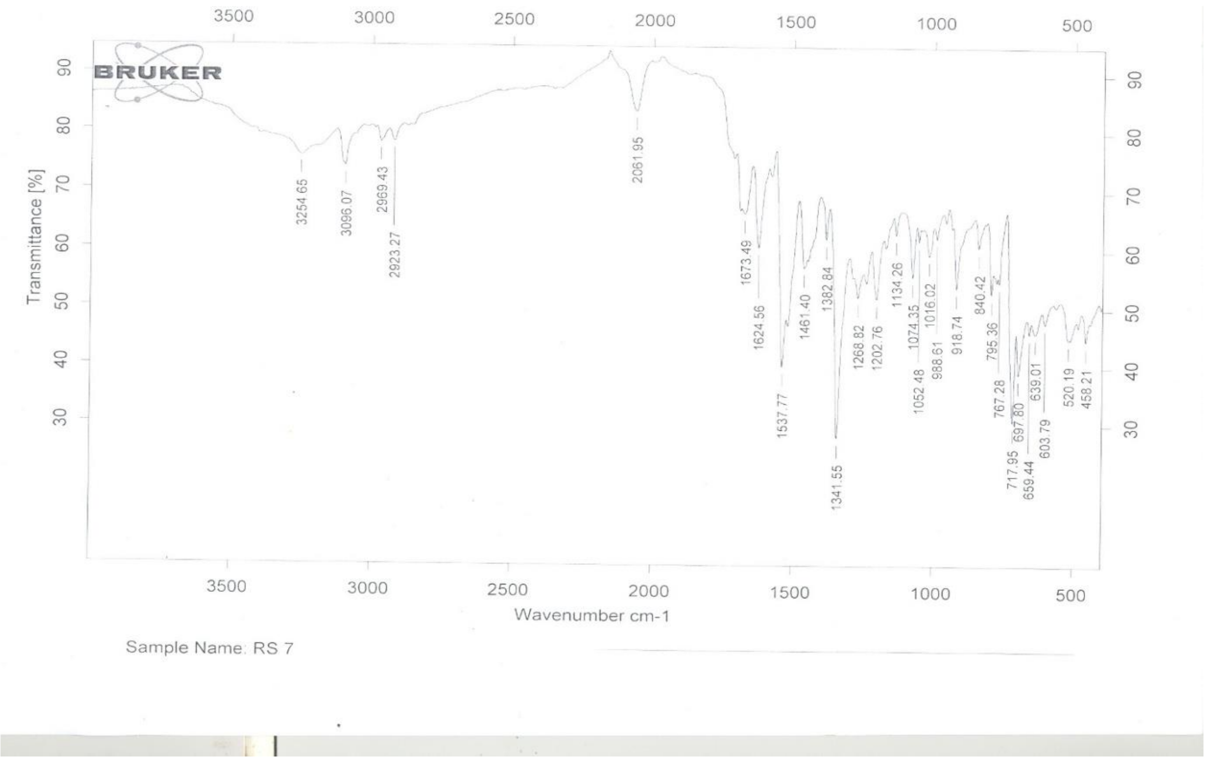

Figure S14 IR Spectrum of **4g**

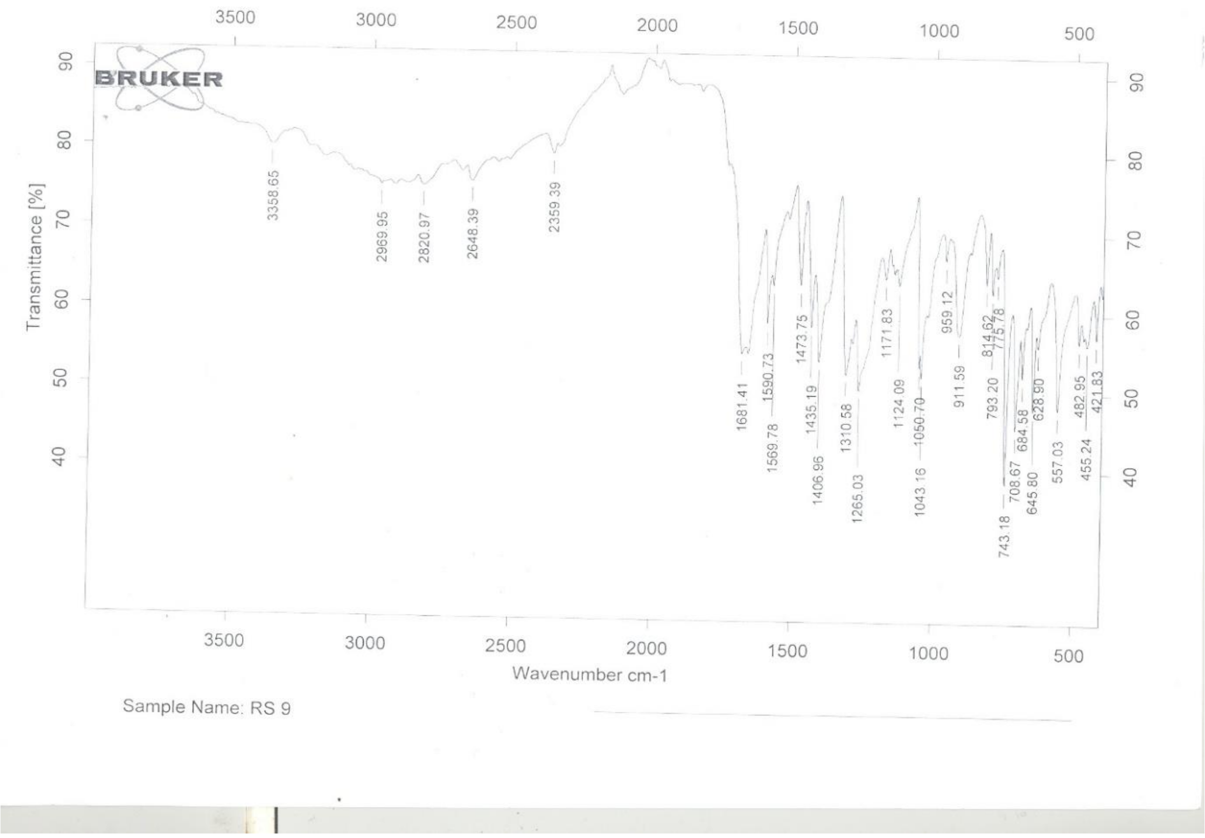

Figure S15 IR Spectrum of **4i**

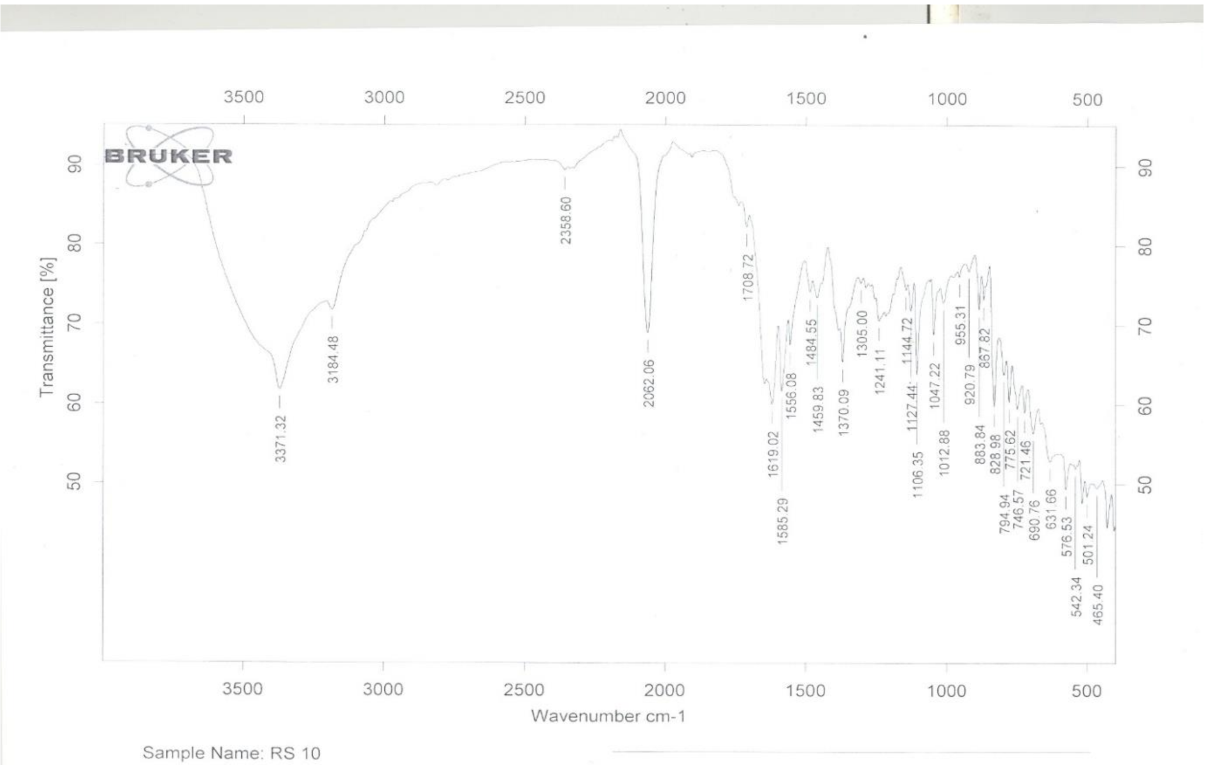

Figure S16. IR Spectrum of **4j**

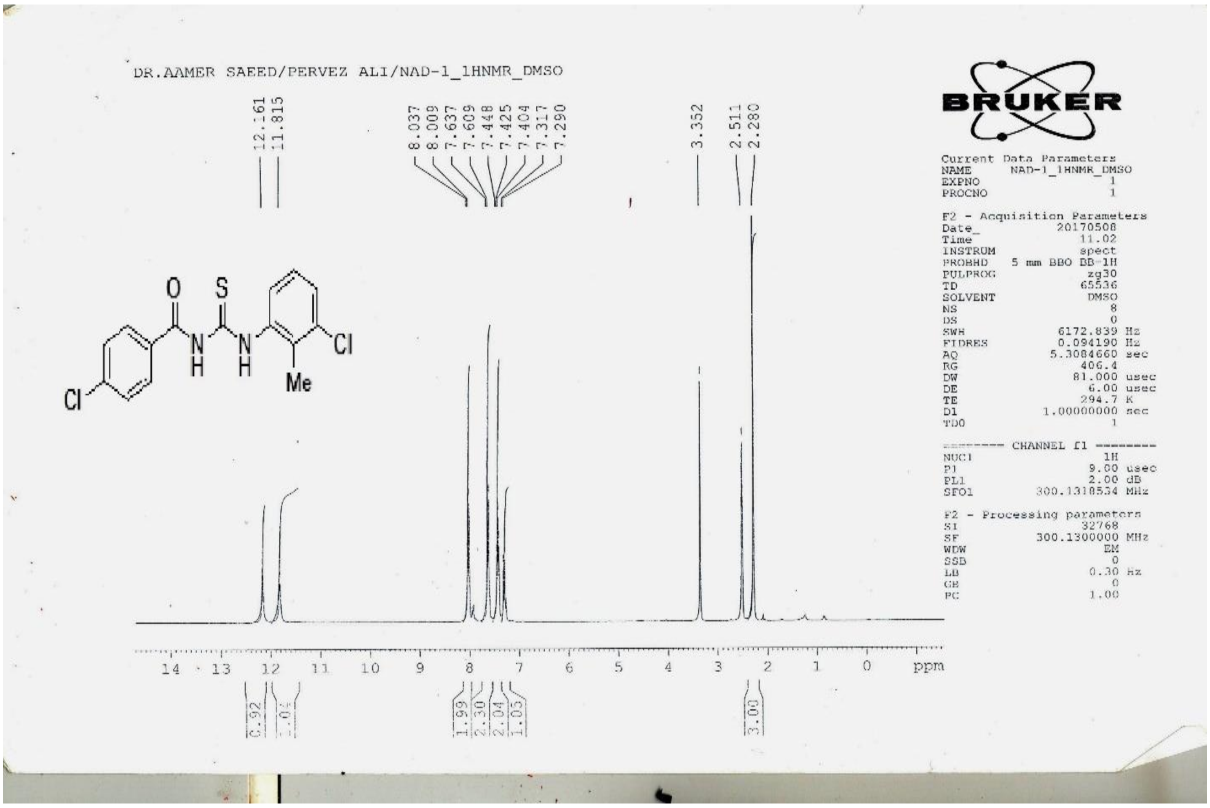

Figure S17. ^{1}H-NMR spectrum of **4h**

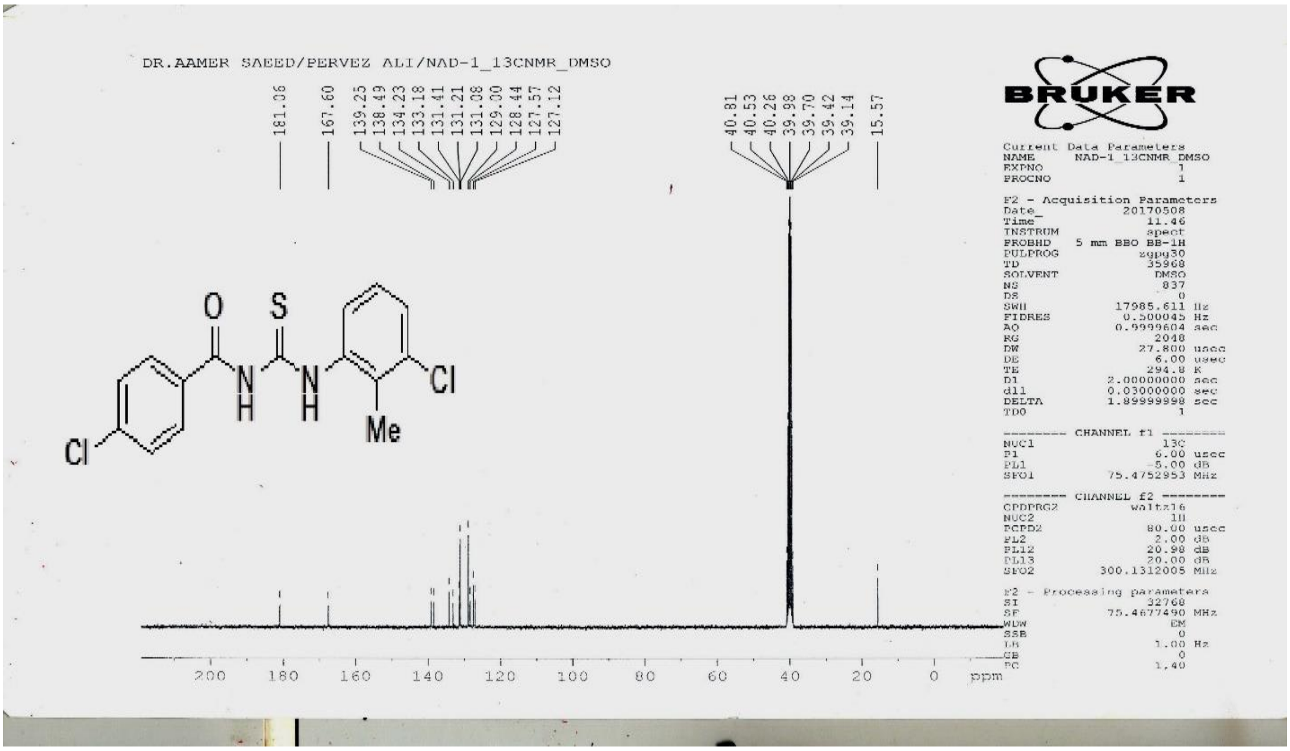

Figure S18 ^{13}C-NMR spectrum of **4h**
